# Public and/or private health care: Tuberculosis patients' perspectives in Myanmar

**DOI:** 10.1186/1478-4505-7-19

**Published:** 2009-07-28

**Authors:** Saw Saw, Lenore Manderson, Mridula Bandyopadhyay, Than Tun Sein, Myo Myo Mon, Win Maung

**Affiliations:** 1 Department of Medical Research (Lower Myanmar), Yangon, Myanmar; 2 Faculty of Medicine, Nursing and Health Sciences, Monash University, Australia; 3 Faculty of Health Sciences, La Trobe University, Australia; 4 National Tuberculosis Programme, Department of Health, Yangon, Myanmar

## Abstract

**Background:**

Tuberculosis is a major public health problem in Myanmar as in other developing countries. About 73% of TB patients seek care at private general practitioners' clinics before presenting to the public TB centre, raising questions about how best to prevent transmission and maintain treatment regimens.

**Method:**

The study was conducted in two townships in Yangon Division in Myanmar in 2004, and examined treatment seeking behaviour of TB patients and their views towards public and private health care services. This was an *exploratory descriptive *study. Both quantitative and qualitative research methods were employed in data collection from TB patients, health care professionals, and members of various agencies involved in TB Control Programme.

**Results:**

A considerable delay was found between the onset of symptoms of TB and seeking treatment (five days – two months). General practitioners were the first point of contact in all cases. Old TB patients influenced the treatment seeking behaviour and choice of treatment clinics of new TB patients. Most patients viewed the public health sector as a place to obtain free treatment and the private sector as a fee-paying, convenient and better place to seek treatment.

**Conclusion:**

The involvement of private general practitioners is crucial for effective TB control in Myanmar. The selection of GPs for partnership with the public sector is vital to the success of public-private partnership in controlling TB.

## Background

Increasing emphasis has been placed in recent years on public-private partnerships in the health sector, in recognition of the current distribution of services and patient choices and preferences, and to support the efficient and effective delivery of health services. Health care provision is considered conventionally to be the responsibility of the public sector and government in developing countries, as reflected in the Alma Ata Health Declaration and other key global mission statements [1]. However, by the mid 1980s, the role of government in health care provision had changed [2], with the World Bank recommending a reduction in the level of government involvement in health care and promoting investment from the private sector. The WHO held a series of meetings from 1990, focusing on the role of the private sector [3-5], and in developing countries, public-private partnerships began to be adopted especially in areas of high need – reproductive health care, diarrhoea, malaria, HIV, sexually transmitted infections (STI) and TB. Many largely inter-related circumstances worked to support the initiation of public-private partnerships (PPP) in developing countries. These included: political and economic changes; the introduction of an open market economy; increased demand by the general public for quality services; health sector reform; limited resources in the public sector; an increased proportion of public staff working also in the private sector; the development and the increased involvement of non-government organizations (NGOs) in health care services; urbanization and diversity of health care providers in peri-urban and slum areas; and poor or sub-standard practices in the private sector.

In developing countries such as Myanmar, privatization was widespread as an unplanned response to the failure of the public sector [2]. Limited human resources and funds in the public sector had led to poor public health services and reduced quality of care especially, but concurrently, the government had not anticipated an increased demand for high technical quality of care and so had no guidelines to interact with the private sector. The support of a market economy and increased demand for quality health care led to an overgrowth of the private sector for infectious as well as non-communicable disease. This included TB, the control of which involves prompt and correct diagnosis and firm adherence to treatment. A series of operational research studies, conducted by the WHO in India, Nepal, Vietnam, Indonesia and Myanmar, confirmed that the private sector was the dominant diagnostic resource even for poor patients [5]. In most member countries in the WHO South-East Asia region, private general practitioners (GPs) manage more than half of *all *tuberculosis patients, despite the fact that diagnostic services, appropriate medication, and monitoring to ensure adherence appear to be ineffective in this sector [6-11].

Myanmar is one of 22 countries globally regarded as having a high burden of TB [12]. According to the National Surveillance Reports produced by the National Tuberculosis Programme in Myanmar, tuberculosis is the fifth leading cause of morbidity and second leading cause of mortality, mainly affecting those in the most productive age groups (15–59) [13]. Annually, an estimated 1.5% of the population is infected with TB, and about 85,000 people progress to develop the disease [14]. In the National Health Plan (NHP 2001–2006), TB is ranked as the third priority disease following HIV/AIDS and malaria. Since 2000, the WHO has encouraged its implementation of public-private partnership projects to improve TB control, and in 2003, the Myanmar Ministry of Health developed guidelines to involve private general practitioners (GPs) in the National Tuberculosis Programme (NTP) [15]. Hence a public-private partnership was initiated, although as elsewhere, there has been limited scholarly attention to the factors that support or discourage its successful implementation and/or sustainability. To find out effective strategies for public-private partnerships, it is important and necessary to take account of both health providers' and patients' perspectives of public and private health care services. In this article, we describe TB patients' preferences and underlying reasons for choosing treatment centres, and analyse their views on public and private health care services.

### Health Services in Myanmar

Health care and medical services in Myanmar are provided by both the public and the private sectors. The public sector, which includes hospitals, diagnostic services, outpatient clinics and primary health care programs, is managed by the national Ministry of Health. The National Tuberculosis Programme (NTP) of the Ministry carries out TB control activities through Township Health Departments (THD) that operate in all 325 Townships in the country. The Township is the unit of management for all disease control activities and is responsible for providing primary health care to an average population of 130,000. The THD provides a laboratory service for sputum microscopy, registers and records TB cases (excluding those registered with private practitioners), maintains drug storage and consistent supplies, and provides anti-TB drugs to patients according to a standardized regimen. THD staff also follow-up people with TB, provide health education to patients and to a lesser extent the community, according to NTP guidelines, and report to the NTP regularly.

In principle, microscopy services for sputum examination are available at the THD, but in many townships this is not the case, and patients may have to visit a District, State or Divisional TB Centre for a sputum examination. One sputum microscopy centre is available per 100,000 people, half of the ideal standard (1: 50,000). Although some midwives have been trained to collect sputum from patients and to send the sample to the district laboratory, patients often have to travel themselves to a district-level TB centre for sputum examination for diagnosis, evaluation and monitoring.

The private sector includes various individuals and institutions, including international and local non-government organizations (NGOs), private general practitioners, private hospitals, private pharmacies, small stationery and basic provision stores stocking medicines, and traditional practitioners. Private GPs have usually trained as general physicians or family medical doctors, but they are a diverse group. They include physicians who are employed in government service but who take private patients outside of office hours, and GPs engaged only in private practice. They also include GPs working in private capacity but registered with an international non-governmental organization (INGO), Population Services International (PSI). As this illustrates, both public-employed and salaried doctors, whether or not they work as doctors in this context, and private doctors who charge consultation fees, run their own private clinics. Most private clinics are open in the evenings; in contrast, government clinics operate from 9 am to 4 pm. At the time of the study, PSI was the only INGO involved in PPP and able to provide free anti-TB drugs, so extending its original role to train GPs to provide reproductive health care (PSI is referred to as INGO in this paper). The Myanmar Medical Association (MMA) also trained GPs for TB control, but at time of the study, it was only involved in encouraging GPs to refer patients to public centres for TB control and to provide health education.

In general, patients in Myanmar prefer to seek care at a GP clinic first irrespective of their socioeconomic status because of their convenient opening hours and their proximity to patients' residences, and the ability of GPs to provide individualized care. However, there is no documented study on the utilization patterns of public and private health care services in Myanmar. We know little about people's selection of a treatment centre for tuberculosis in Myanmar, and there has been limited work on choice of treatment centres for TB patients [16]. One social science study on the burden of TB, and a quantitative study on treatment seeking of TB patient [17-19], showed that 73.3% of TB patients sought care at GP clinics in preference to a public facility as first action in treatment seeking. In addition, a pilot study in Kyauk Se Township, conducted by Department of Medical Research (Upper Myanmar) in collaboration with the Myanmar Medical Association (MMA) and the National Tuberculosis Programme (NTP), illustrated the value of involving private GPs in TB control: Private GPs contributed 44% of new smear positive cases registered over a two-year period (2002 to 2004) [20]. The notification of new sputum smear-positive TB in this Township increased by 85% from 46 to 85/100 000 from July 2002, the year prior to GP involvement, to December 2004 (2 years later). The treatment success rate for new smear-positive cases treated by GPs was 90%, leading the authors to conclude that the involvement of private GPs substantially increased TB case notification and maintained a high treatment success rate [20].

The present study on TB patients' treatment seeking preferences was undertaken to explore their reasons for choosing treatment centres, and to elicit their views on public and private health care services in Yangon.

## Methods

### Study design

The study reported in this paper is part of a larger study that was conducted with the aim of exploring how the public-private partnership currently operates in Myanmar, identifying constraints to efficient and effective operations of the partnership, and on this basis identifying possible strategies to improve public-private partnership in Myanmar. Both qualitative and quantitative methods were used in data collection. In this paper we report only the qualitative aspects of the study, which used exploratory descriptive methods, particularly, face-to-face interviews. In-depth interviews were conducted with TB patients who had presented to and were being treated by doctors in the public sector (i.e. through the Township Health Department) and who were being treated in the private sector, through GP clinics, in Yangon. Twenty key informant interviews with National Tuberculosis Programme staff and TB coordinators from Township Health Departments were conducted. Qualitative methods enabled the exploration of complex areas not amenable to quantitative research [21-23].

### Study area

Yangon Division is located in south or lower Myanmar. It has a population of 6,187,685, including residents of the capital city, Yangon, and 45 townships situated in the Division. Since Yangon is the capital city, private sector involvement in health services and the provision of care is quite high. Private GP clinics are particularly abundant, with about 50 to 100 GP clinics in an urban township (with a population of 50,000 to 200,000) compared with only about three to five in a village tract.

For the study from which we draw, two townships in Yangon Division were selected on the basis of the following criteria: that a Township Medical Officer acted as a TB team leader, there was a District TB team or NTP staff responsible for TB control, and the townships were located in peri-urban areas of Yangon.

### Data collection

Both female and male TB patients (21 male, 10 female) were recruited from the THD and GP clinics for inclusion in the study on a continuing basis, without specific selection criteria other than age ≥ 18 years. All registered TB patients who were under treatment (that is, both new and old TB patients) were included. We did not specifically choose old or new TB cases, but as interviews progressed our analyses showed that some of the interviewees had TB previously (hence we classified them as 'old cases') and patients who contracted TB for the first time were classified as 'new cases.' Since we were exploring TB patients health seeking behaviour specifically – which incidentally was very similar for both groups of patients – we did not treat them as separate group for analytical purposes. The pathway for old or new cases had common elements and we were interested in exploring these further.

TB patients were recruited through receptionists from the THDs and all three categories of GP clinics – (a) GPs employed in government service part-time and saw patients privately outside of office hours, (b) GPs engaged only in private practice, and (c) GPs working in collaboration with the INGO involved in TB care in Yangon. In Myanmar, most GP clinics do not have receptionists or assistants. It was not possible to recruit from the waiting area because of privacy and confidentiality issues. We had no knowledge of patients' TB status, as we could not access patients' register or records without their consent. Enlisting the attending GP's help was the only recourse we had to recruit patients for in-depth interviews. GPs were instructed not to advise patients nor coerce them to participate in the study, but to provide general information and to suggest to them that, if interested, they contact the researcher.

Recruitment continued until the diversity of experience was identified and understood [24], i.e., no new information was emerging from the interviews after initial analysis of the data, or from later complete analysis which was conducted in the field. As we reached data saturation with 31 patients, i.e., we did not obtain any new additional information, we did not recruit any more patients for in-depth interviews. Interviews were based on a pre-tested interview guide. The interviews lasted for about one hour to 90 minutes.

An interview guide was used and interviews were tape recorded with the permission of interviewees. All interviews were conducted in Burmese by SS. Each person was interviewed once only. During data collection, emerging issues were identified that were explored in subsequent interviews. Reflexive notes and field observation notes were also made (by SS and MB) to assist in the interpretation of qualitative data.

### Data analysis

The main themes for interviews were derived from the study objectives to explore reasons for choosing private or public and patients' views on public and private health services in Myanmar. Hence the semi-structured in-depth interview guide covered three main themes – treatment seeking, choice of treatment (public or private), and opinions and suggestions related to the public-private partnership.

The interviews were transcribed and organized on the basis of emerging themes and sub-themes. SS transcribed and read over the transcripts to identify primary themes, from the interview guide and from the data before organizing data with ATLAS ti (version 5) software. Main themes and emerging themes, and their relationship to each other, were explored, and the inter-relationship of themes was summarized for discussion with the other authors. Sixty five codes were identified; some codes were merged after detailed analysis. Primary code families were organized and linkages and relations among codes and code families were observed. This facilitated the analysis and interpretation of the qualitative data.

### Ethics

Prior to conducting this study, we acquired ethical clearance from relevant institutes including the Ethical Committee on Medical Research involving Human Subjects from the Department of Medical Research, Lower Myanmar; the Research Ethics Review Committee from the World Health Organization, Geneva; and the Human Research Ethics Committee from The University of Melbourne. The participants (BHS, NTP staff and GPs) were de-identified and were assigned a number, and quotes are attributed on this basis (e.g. BHS 1, NTP staff 1, GP 1). In quoting patients, gender, age and treatment centre (THD or GP clinic) are used for identification: For instance, a 28-year-old man who was receiving treatment at the PSI GP clinic is identified as "M 28 treated at PSI GP clinic."

## Results

As noted above, 21 male and 10 female patients were recruited, reflecting patient distribution in Myanmar (2:1). The ages of respondents ranged from 24 to 78 years (mean = 41 years). Almost all respondents were labourers, although a few were university students, unemployed or otherwise not in the paid workforce. Fourteen patients were seeking care at the Township Health Department (public sector) and the remaining 17 were seeking care from GPs at time of interview.

### Treatment seeking behaviour

New patients reported that they were motivated and encouraged by former patients, who they knew had been cured of the disease. Former TB patients, including relatives, friends and neighbours of newer patients, provided information on diagnosis and treatment, advising them to seek care in the public health sector or from a private GP clinic where free treatment was available. A few patients were advised to go to public sector by a local administrative officer or an employee of the urban health centre. Township Health Department and INGO GP clinics were the most common places of referral by former patients.

**Figure 1:**
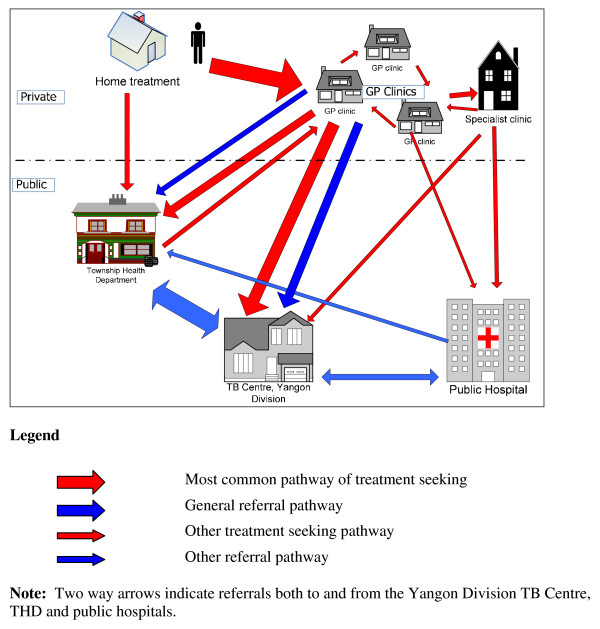
Treatment seeking among TB patients.

The most common pattern of treatment seeking is shown in Figure 1. Although almost all TB patients sought health care treatment at GP clinics first, most had already used herbal medicine or other home remedies to relieve coughing symptoms. Twenty four out of 31 patients took medicine at home before seeking care at GP clinics or public sector. Almost half of them tried traditional medicine to relieve cough and fever. Close relatives suggested different kinds of home remedies and herbs. Eight patients stated that they tried western medicine such as paracetamol, cough syrup, cold tablets and other antipyretics. One patient revealed that he had bought an over-the-counter anti-TB drug (AKT-4, a combination of Rifampicin, Isoniazid, Ethambutol and Pyrazinamide) at a drug store and had self-medicated. He had contracted TB four times. A few patients took both western and traditional medicines at home. There was a wide range in the duration reported by study participants of taking home treatment, from 2–3 days to one year:

At first, I though I had flu. So, I took some western medicine like Biogesic for a week. I took *Aung Ta Man *(traditional medicine for fever, packed in powder form) too. Usually, I get relief after taking a couple of packets. But this time, there was no relief after a few days. Then I went to see the GP. (M 41 treated at THD)

Table 1 shows the steps taken by 25 patients who first sought care outside the home at GP clinics, before laboratory-confirmed diagnosis with TB. Of the remaining six, one went straight to the TB centre, Yangon Division, four went to a specialist clinic and one went to the Township Health Department.

**Table 1:** Treatment seeking behaviour before diagnosis with TB

**Order**	**Domain and strategy**	**Purpose and action**
1	Home treatment	Trial with home remedies and herbs

2	Self-medication	Use of western and traditional medicines from over-the-counter stores

3	Sought advice from friends and relatives	Advice from friends and relatives influenced treatment seeking patterns and choice of treatment centre. Old TB cases provide advice informally to new TB cases

4	Seeking treatment from GPs as first formal source	All patients first sought care at GP clinics

5	Shopping around treatment centres	Some patients opted to see different GPs and specialist clinics when symptoms did not subside. Some patients sought care at more than one treatment centre.

6	Seeking care from public health service	Patients went to public health service when their symptoms did not improved after seeing many GPs or when they suspected themselves of contracting TB

Eight participants continued to take treatment at the GP clinic at which they first presented. The rest (23 out of 31) sought care at more than one clinic because symptoms were not relieved by the treatment prescribed at the first clinic. A few patients (3 out of 31) were hospitalized and after discharge were referred to the THD. It was also quite common for TB patients to consult with a number of health care providers during the course of their illness. The main reason for switching from one clinic to another was because they felt that they were getting no relief from persisting symptoms.

I was coughing for about 20 days and had fever for 15 days. I ignored it and did not take any care or treatment for this condition. But, I had no appetite and was losing weight. Then I went to an *inn-galaik say-khan *(western medical clinic), the doctor said that my lungs were bloated. I took treatment there for three days and took my medication regularly, but I was still coughing. Since the treatment was not effective I went to another GP clinic and again took treatment there for three days.

Unfortunately, my symptoms were still persisting, and I lost a huge amount of weight. So I rested at home for about four to five days without seeking any treatment from any clinic. At that time, my sister-in-law visited me and said that she suspected TB, because when she contracted TB, she too felt the same. She advised me to go and seek care at *Aung San *[TB Centre, Yangon Division]. Finally I went there and was diagnosed with TB. (F 44 treated at THD)

The main reason for switching from GP clinics to the public sector was the financial constraints associated with long-term treatment. The average consultation fee per visit at GP clinic is about 3000 *kyats *(US $3) and the cost for anti-TB drugs is also 3000 *kyats *per day. Cost of anti-TB drugs for the full course (i.e. six months) is 84,000 *kyats *(US $840). While patients need to purchase drugs and pay consultation fees at GP clinics, TB treatment is available free in the public sector, including from both the TB Centre and the Township Health Departments. Most patients got anti-TB drugs from the Township Health Department (THD) and consulted with their respective GPs to relieve other symptoms of TB, with some patients sought treatment from both the private and public sector simultaneously. This is well illustrated by a 55-year-old participant taking treatment at the THD.

When I got fever, I went to the GP clinic in my ward. *Sayama *(lady GP) told me that I got *asoak pwa *(inflamed lungs) and then prescribed drugs. She did not tell me that it was TB. I took the drugs for 16 days. Afterwards, I sought care at another GP since the symptoms were not relieved. The second GP was lung specialist. He told me that I had not contracted TB. Then I stopped taking treatment. After a couple of months, I coughed with blood. So, I went to a third GP clinic where I am currently taking treatment. (M 54 treated at GP clinic)

The finding – that patients prefer the GPs to whom they usually present but finally end up at THD for economic reasons – is consistent with the findings from in-depth interviews with GPs and BHS:

When we refer, few patients refuse to go to the THD. We have to explain that drugs are provided for free, and more effective treatment is available at THD, etc....Even then, they prefer their family GP...TB mainly affects the poor and they cannot afford long term treatment at private GP clinics. So I always refer my patients to THD (In-service GP 2).

### Public and/or private care?

The international NGOs, Population Service International (PSI), as noted above, has been involved in TB control in collaboration with the NTP since 2003. According to the Memorandum of Understanding (MOU) between the Ministry of Health and PSI, a PSI GP can charge a consultation fee of 300 *kyats *(US$ 0.30) for one visit, but anti-TB drugs have to be given free. At the time of the study, three INGO clinics operated in both study townships, providing health care services for reproductive health and sexually transmitted diseases, TB treatment and general family medicine. Among those taking treatment at outpatient clinics at THD (13), five indicated their preference also to go to GP clinics. All patients receiving treatment at INGO GP clinics reported that they were satisfied with the service; these people all had prior experience of attending INGO clinics for minor ailments. Therefore, once diagnosed with TB, they elected to receive treatment from these clinics rather than going to the public clinic or attending their own, fee-for-service, GP. However, all patients (18) who were treated at GP clinics stated that they preferred their family doctors (GPs), even when their GPs were not affiliated with the INGO and so were unable to provide free anti-TB drugs; despite the costs, they preferred this option for as long as they could afford to do so, because of their familiarity with and trust in their provider.

A fuller exploration of choice of treatment centre indicated that preferences are related in part to distance from home and/or workplace, opportunities to ask questions and to receive clarification, the ability to receive concurrent treatment for other diseases such as Diabetes Mellitus, prior acquaintance with the GP, and cost (hence the choice of PSI clinics). Patients receiving treatment at GP clinics and those at the THD had similar views on the public health care service in terms of opening hours, long waiting times and complicated procedures: A few patients also mentioned the inconvenience of paper work at THD that was necessary for them to get free treatment, and for this reason, they switched to a GP clinic. Some patients were seeking care for TB at more than one centre, but there was no communication between GP clinics and the THD to establish which patients were receiving treatment from more than one clinic.

I do not go to the Township Health Department because it would take too much time. For us, time is money. I have to work for my day-to-day living and cannot spare working hours to attend THD. (M 32 treated at GP clinic)

If the cost of treatment were the same, I would go to a GP clinic, because it's more convenient for me. It's close to my home and I could go in the evenings. (F 33 treated at THD)

Some patients regarded THD as a high-risk public place, too, where one could contract severe infections from others. Participants described THD as overcrowded, with dark and narrow spaces where they could easily get infected. A few patients pointed out that they were afraid that if their relatives were to accompany them, they would contract TB from other severely ill patients at the THD. These ideas of risk, contagion and stigma, that inhibited people from reporting disease and influenced family and community interactions, were widely held:

At Township Health Department, if there are a few patients, it is convenient and you don't have to wait too long. But if there is a long queue, I worry that we might contract severe disease from others while we're waiting there. All sorts of severe cases are mixed up in the crowd. (F 42 treated at GP clinic)

Yet much of the reasoning by patients was contradictory. In both townships, THDs are close to residential areas and can be reached by bus within a half an hour. Although most patients receiving treatment at the THD stated that they did not have any problems of access, all of them had visited a GP clinic first. When cost became a major concern for those requiring long-term treatment, patients switched to THD where they could get free drugs. There is considerable uncertainty and distrust by patients about GP's willingness to provide free treatment at their clinics. Most patients saw GP clinics as a commercial venture – a place to pay – and the THD as a place where they could get free treatment, as illustrate below:

What do you think about collaboration between GP clinics and the public sector like THD?

Well, GP clinics are for well-off people. You don't have to wait for long there whereas we have to wait at THD. But it's free at THD and I don't think we can get free drugs at GP clinics.

What if the government were to distribute drugs free to GP clinics?

(Laughing) It's hard to say. If the government forces GPs to provide free drugs, that may be possible, I suppose. It depends on individual doctors, I would say. Some GPs wouldn't be able to do so and might have to charge for drugs. It would be hard for patients if the GP were to ask for money. I think our *Sayama *(the lady GP) could collaborate with THD. She is kind; also she lives in our suburb and is close to and friendly with all of us. However, there are different kinds of people (working as GPs). Some may not want to be bothered with (TB) patients. Moreover, some people might be reluctant to go to a GP clinic for free treatment, because GP clinics are regarded as places where you have to pay to get treatment. (F 26 treated at GP clinic)

The best thing is to provide free drugs through GP clinics that are convenient for patients. Then, time and money can be saved. Yet it depends on the individual GP – how much he or she would contribute in terms of their time. (M 55 treated at GP clinic)

Patients receiving treatment at GP clinics had a different view of their family GP and other GPs. They trusted their own GPs to provide free drugs if the drugs were supplied by the public sector. However, they indicated that the contribution by GPs to TB control would depend on the individual GP. According to patients, some GPs might not be interested or willing to collaborate with the public sector, for financial or other reasons. Most patients stated that it would be more convenient for patients if they could get free treatment from any GP clinic, but they felt it neither realistic nor practical to expect all GPs to collaborate.

It would be great if anti-TB drugs were distributed to all GP clinics. But, I'm only sure about our *Saya *(the GP), who is very kind and good towards his patients, you know. It mainly depends on the individual, whether he would give free treatment or not. (F 23 treated at one of the INGO GP clinic)

I think it is possible to distribute free drugs through all GPs in our suburb. But, I don't think specialist clinics would do so. Specialist clinics are always busy and hunting for money. Therefore, providing free drugs and specialist clinics are like east and west. (F 26 treated at GP clinic)

Some patients mentioned that GPs would not be willing to contribute unless they were obliged to do so; they felt that some GPs would be reluctant to spend clinic hours with TB patients without being paid. A few also pointed out that collaborations between public and private were not sustainable because GPs were not responsible for providing public health services such as providing free treatment to TB patients. However, all patients agreed that a public-private partnership was a good idea and would benefit them:

Collaborations between GP clinics and THD are not always easy in the long run. It would be good if a GP has *cetanar *(good will) towards patients. Public staff have the responsibility of taking care of TB patients, and they are obliged to provide care, whereas GPs have no obligation. I think that if GPs do not get any benefit from it in the long run, they'll be disappointed and will pull out from collaboration. (M 42 treated at THD)

## Conclusion

Participants reported considerable delay between the onset of symptoms of TB and seeking treatment. Few regarded cough alone as a serious symptom warranting medical attention, and so did not suspect TB initially. Once diagnosed, although all study participants were aware that they could get free treatment in the public sector and viewed this positively, a number opted to seek care at a private GP clinic, at least in the short term. Consistent with findings from other studies conducted in the South East Asia region, the GP was the first point of contact for all patients [25-27]. Cost was a significant reason for switching to or consulting with public providers, especially because TB is known as requiring long-term treatment. Although all patients sought care at GP clinics first, they had little confidence and/or trust in GPs to give them free treatment. The participants viewed GP clinics as places at which they would have to pay for services, and saw the THD and public health care services as the appropriate places to get free treatment. As cost was a major concern for most study respondents, their preference for private health care is not easily interpreted. Further, among those who were receiving treatment at the THD, a number indicated that if the cost were the same, they would prefer to see a GP. Common elements were observed in health seeking behaviour pattern amongst the study population irrespective of age, geography, or poverty.

The reluctance of participants to attend the THD for any reason other than access to free drugs, and the preference to remain in the care of their own family practitioner rather than an INGO GP or the THD, highlight the potential for and difficulties associated with public-private partnerships. We did not explore whether there was a difference in adherence to treatment and successful cure, according to provider and attitude to services. The strength of views and preferences suggest, however, that access to and relationships with providers may be significant in the effectiveness of TB treatment, indicating that selection of providers in PPP is crucial for the programme's success. On the basis of this study, the NTP and MMA have identified specific selection criteria for GP involvement in PPP:

1. Providers who have many TB patients under their care (on the presumption that patients will prefer to see that specific provider); and

2. Providers who diagnose and treat TB patients provide free TB drugs and follow-up patients.

It is too soon to comment on the feasibility of the PPP programme as it is still in its infancy.

## Competing interests

WM is a Program Manager and Deputy Director a the National Tuberculosis Programme, Myanmar. The remaining authors declare that they have no competing interests.

## Authors' contributions

SS developed the research proposal, conducted the data collection, and undertook the data analysis and interpretation of data in fulfilment of her PhD studies. LM and MB supervised the research and worked with SS to develop instrumentation, and to analyse and interpret the data, and write the dissertation. TTS, a local supervisor, provided technical and administrative support during the field work. MMM assisted in data collection and interpreting the data. WM, a programme manager of the National Tuberculosis Programme, provided technical advice and administrative assistance and coordination for the field work. All authors have read and approved the final manuscript.
